# Genetic Alterations in Gliomas Remodel the Tumor Immune Microenvironment and Impact Immune-Mediated Therapies

**DOI:** 10.3389/fonc.2021.631037

**Published:** 2021-06-08

**Authors:** Maria B. Garcia-Fabiani, Santiago Haase, Andrea Comba, Stephen Carney, Brandon McClellan, Kaushik Banerjee, Mahmoud S. Alghamri, Faisal Syed, Padma Kadiyala, Felipe J. Nunez, Marianela Candolfi, Antonela Asad, Nazareno Gonzalez, Marisa E. Aikins, Anna Schwendeman, James J. Moon, Pedro R. Lowenstein, Maria G. Castro

**Affiliations:** ^1^ Department of Neurosurgery, University of Michigan Medical School, Ann Arbor, MI, United States; ^2^ Department of Cell and Developmental Biology, University of Michigan Medical School, Ann Arbor, MI, United States; ^3^ Immunology graduate program, University of Michigan Medical School, Ann Arbor, MI, United States; ^4^ Leloir Institute Foundation, Buenos Aires, Argentina; ^5^ Instituto de Investigaciones Biomédicas (INBIOMED, UBA-CONICET), Facultad de Medicina, Universidad de Buenos Aires, Buenos Aires, Argentina; ^6^ Department of Pharmaceutical Sciences, University of Michigan, Ann Arbor, MI, United States; ^7^ Biointerfaces Institute, University of Michigan, Ann Arbor, MI, United States; ^8^ Department of Biomedical Engineering, University of Michigan, Ann Arbor, MI, United States

**Keywords:** glioma, immune microenviroment, immunotherapy, mouse model, clinical trial

## Abstract

High grade gliomas are malignant brain tumors that arise in the central nervous system, in patients of all ages. Currently, the standard of care, entailing surgery and chemo radiation, exhibits a survival rate of 14-17 months. Thus, there is an urgent need to develop new therapeutic strategies for these malignant brain tumors. Currently, immunotherapies represent an appealing approach to treat malignant gliomas, as the pre-clinical data has been encouraging. However, the translation of the discoveries from the bench to the bedside has not been as successful as with other types of cancer, and no long-lasting clinical benefits have been observed for glioma patients treated with immune-mediated therapies so far. This review aims to discuss our current knowledge about gliomas, their molecular particularities and the impact on the tumor immune microenvironment. Also, we discuss several murine models used to study these therapies pre-clinically and how the model selection can impact the outcomes of the approaches to be tested. Finally, we present different immunotherapy strategies being employed in clinical trials for glioma and the newest developments intended to harness the immune system against these incurable brain tumors.

## Introduction

Malignant tumors of the central nervous system (CNS) have an annual rate mortality of 9.01 per 100,000 adults in the US (1). Gliomas are brain tumors which clinically can present as grades II–IV in relation to their malignancy. Glioblastoma, the most aggressive type of glioma (high-grade glioma, WHO grade IV), accounts for the majority of gliomas and the highest incidence rate for malignant tumors of the CNS in adults (3.21 per 100,000 population) (1). This type of aggressive tumor has been subjected to extensive research due to the dismal outcomes of the current standard of care (SOC) therapies (maximal safe surgery, followed by radiation and chemotherapy with Temozolomide), and the lack of improvement in the median survival post-diagnosis (14-17 months) (2).

There are several aspects of this type of tumor that makes it difficult to treat (3), such as its anatomical location and the presence of a blood-brain barrier, which hampers the delivery of therapeutics (4); its intrinsic infiltrative nature, that makes it a tumor virtually impossible to resect completely (3, 5); and the presence of an immunosuppressive micro-environment, that impedes the natural development of an anti-tumor immune response (6–11). In spite of these challenges, in the last decade, there has been an expansion in the therapies aimed to harness the immune system to direct it against malignant glioma (12). So far, pre-clinical data has demonstrated the effectiveness of immune-stimulatory or anti-immunosuppressive strategies, and many clinical trials are currently ongoing to test their efficacy in the clinical arena (12).

This review aims to discuss several aspects related to the glioma immune-microenvironment and the newest strategies that could emerge as a result of the latest pre-clinical investigations. Firstly, we will present the available clinical data regarding the immune microenvironment in glioma and its particularities in terms of tumor classification and molecular features (7, 13–15), as well as the current immune-mediated strategies being tested in the pre-clinical field (16). Also, we will overview the present immune-stimulatory therapeutic modalities being tested in clinical trials (8, 17). Finally, we will discuss the latest pre-clinical developments related to anti-glioma therapies that could enhance the immune system to develop long-lasting anti-tumor immunity (18–24).

We believe that this review will bring to light the latest improvements in the strategies being developed to treat high-grade gliomas aimed to stimulate an anti-tumor immune response, broadening the spectrum of possibilities to be tested in the clinical setting and bringing new concepts for fighting this devastating tumor.

## Glioma Classification

### Adult Gliomas

Glioma involves a heterogeneous group of primary brain tumors originated from neural precursor cells (25), and represent thirty percent of the CNS tumors (1, 26). They can be divided in diffuse gliomas and non-diffuse gliomas, which refer to tumors with a circumscribed growth pattern, including ependymomas and other astrocytic tumors (27, 28). The majority of adult gliomas are diffuse, distinguished by an infiltrative pattern of growth within the CNS parenchyma, and have been typically classified according to histological features and grade of malignancy (27–29). The histological analysis of surgical specimens allows the identification different glioma subtypes: oligodendroglioma, characterized by uniformly rounded nuclei; astrocytoma, with nuclear irregularities and hyperchromasia; and oligoastrocytoma, which is a rare mixed glioma (30). Additionally, based on the grade of anaplasia it is possible to further divide gliomas into four World Health Organization (WHO) subtypes, ranging from WHO grade I to WHO grade IV. WHO grade I gliomas correspond to tumors with slow development and better prognosis; WHO grade II gliomas are defined as low grade gliomas; WHO grade III gliomas are used to describe anaplastic gliomas; and WHO grade IV encompass glioblastoma (27, 28, 31). Usually, high grade gliomas (HGG) include WHO III and IV gliomas.

The revised 2016 WHO CNS classification includes, for the first time, distinctive genetic/epigenetic alterations to define several groups of gliomas (28, 32). The presence and distribution of genetic alterations in brain tumors, such as alterations in *PI3K, PDGFR, PTEN, TP53, IDH, EGFR, H3F3A, ATRX* and *TERT* (33–35), are now a criteria used to differentiate glioma subtypes (28, 36, 37). Each molecular glioma subtype is related to a histologic tumor-class and a particular WHO grade of malignancy (33, 34, 38–40). The hallmark genetic alteration in adult diffuse gliomas, that promoted the incorporation of molecular features in their classification, is the mutation in isocitrate dehydrogenase 1 (IDH1). This alteration, usually at arginine 132 (IDH1-R132H), is highly frequent in diffuse low-grade gliomas (LGGs; WHO grade II), in anaplastic astrocytomas (WHO grade III), and also in a smaller proportion of HGG originated from LGGs (secondary glioblastomas; WHO grade IV) (28, 40–42). IDH1-R132H (mIDH1) catalyzes the production of 2-hrydroxyglutarate, eliciting epigenetic reprogramming of gene expression (33, 40, 43, 44) and is associated with better prognosis in glioma patients (33, 39, 40, 45). In addition, the loss of 1p/19q chromosomal segments define mIDH1-1p/19q-codel and mIDH1-noncodel glioma subtypes. Mutant IDH1-noncodel typically co-occurs with loss-of-function mutations in *ATRX* and *TP53* genes, which are associated with astrocytoma and oligoastrocytoma subtypes (28). Mutant IDH1 1p/19q-codel gliomas are usually oligodendrogliomas and frequently co-express mutations in *TERT* promoter (*TERT*p) and *CIC* (28, 39–41). In adults, diffuse wild type (wt) IDH1 gliomas appear principally in patients over 50 years old and commonly are HGG, WHO grade IV of malignancy (28, 31, 39). These HGG generally harbor mutations in *TP53* and *TERT*p, with retention of ATRX function. They can also present alterations in the chromosomes 7 and 10, deletions in *CDKN2A/B*, and changes in genes involved in the RTK-RAS-PI3K signaling cascade, such as *PTEN* mutation or loss or EGFR amplification (28, 31, 32, 34). Importantly, the DNA methylation, which typically occurs at cytosines followed by a guanine separated by a phosphate group (CpG site), emerges as a distinctive parameter to refine tumor classification with clinical implications, especially in cases with ambiguous histology. The CpG-island methylator phenotype (G-CIMP) is closely related with IDH1 mutation and is associated with better prognosis in gliomas (46, 47). On the other hand, demethylation in CXCR4, TBX18, SP5, and TMEM22, genes have been linked with initiation and progression of glioblastoma (48). DNA methylation profiling has been shown to be highly robust and reproducible. In diffuse glioma TCGA patients, Ceccarelli et al., identified glioma DNA methylation clusters (LGm1–LGm6) linked to different molecular glioma subtypes (40). More recently, Capper et al, developed a DNA methylation-based classification system, which allowed to define five categories of methylation classes of CNS tumors, which resulted in a change of diagnosis in up to 12% of prospective cases analyzed (49); and in the positioning of this method as a powerful tool to improve glioma classification. In addition, the analysis of DNA methylation profiles has utility in therapeutic decisions. The presence of methylated CpG islands in the O6-methylguanine-DNA methyltransferase (MGMT) promoter is a molecular marker of better response to DNA alkylating agents (50), indicating that the methylation status of MGMT promoter is a critical feature to design glioma treatment.

In summary, adult gliomas are classified by histological features and by molecular lesions, that define distinctive tumor entities, which are associated with different grades of malignancy. This classification is relevant for diagnosis, prognosis and clinical decisions. In addition, the updated CNS-WHO classification for brain tumors is a valuable source to improve and conduct accurate studies of gliomas, considering the intrinsic biological features of the different glioma subtypes.

Currently, the adult glioma SOC includes maximal safe surgery when is possible; chemotherapy, generally with temozolomide (TMZ); and focal radiation (17, 51). However, in spite of intense investigation for years, no substantial clinical improvements have been observed (51). This unfortunate fact encourages the development novel therapeutic approaches for a wide spectrum of glioma patients who are waiting for an effective treatment.

### Pediatric Gliomas

High grade gliomas comprise ~ 15% of all central nervous system (CNS) pediatric tumors (52), and have an incidence of approximately 0.85 per 100,000 children (26). Pediatric high grade gliomas (pHGG) and diffuse intrinsic pontine gliomas (DIPG) (recently included into the classification of Diffuse midline glioma (DMG)) are highly aggressive gliomas, which, unlike the adult counterparts, occur throughout the CNS anatomy. The prognosis for pHGG is dismal, with an overall median survival of 9-15 months and a 5-year survival rate of less than 20% (53).

Brainstem gliomas are more prevalent in childhood, whereas hemispheric pHGG, are more prevalent in adolescents (54). Several characteristics distinguish pHGG from adult gliomas, such as molecular (genetic and epigenetic), and clinical features (55). Particularly, advancements in molecular high-throughput profiling over the last few years improved our understanding of pHGG and led to the identification of unique genetic and epigenetic features of these tumors. Most notably, the discovery of recurrent mutations in the genes encoding histone variants H3.3 (*H3F3A*) and H3.1 (*HIST1H3B/C*), and other genes associated with epigenetic mechanisms, demonstrated the unique biology of pediatric brain tumors (53, 56, 57). Three somatic mutations resulting in the replacement of a lysine with a methionine at residue 27 of histones H3.1 and H3.3 (K27M) in brainstem/midline pHGG, or the replacement of a glycine to arginine or valine at residue 34 (G34R/V) of the histone H3.3 in hemispheric pHGG were found to be characteristic of these tumors (53, 57). These mutations rewire the epigenome, resulting in global hypomethylation and disrupt critical regulatory sites of post-translational histone modifications (56). These mutations are exclusive, are found at specific anatomical locations, within distinct age groups and patients harboring these tumors have different survival outcomes (38, 56).

The WHO classifies pHGGs as anaplastic astrocytoma (WHO grade III) and glioblastoma (GBM; WHO grade IV) (28). Among midline pHGG, the updated 2016 WHO classification of tumors of the CNS classifies the DMG H3-K27M-mutant as an independent entity, WHO grade IV (58). DMG H3 K27M-mutant arises in all midline CNS structures, are astrocytic tumors, and represent the majority of infiltrative brainstem glioma (59).

The histological characteristics of pHGG include hypercellularity, nuclear atypia, abnormally high mitotic activity, and increased angiogenesis and/or necrosis, the latter two associated primarily with GBM morphology (60). Due to their proliferative nature, HGG have shorter duration between symptom onset and diagnosis compared to tumors of lower grade, precluding the clinical advantages of early detection (61, 62). Surgical intervention of non-brainstem pHGG patients includes tumor resection and biopsy, although total tumor resection is often impossible in pHGG, particularly for midline pHGG, as these infiltrative tumors often progress into normal tissue beyond surgical margins (58). However, the extent of resection is one of the few significant prognostic markers for overall survival (OS) in pediatric patients with pHGG (63). Although surgery is the primary intervention for treatment of non-brainstem pHGGs, it is not curative. Standard of care also includes radiation therapy for pHGG patients above three years of age, typically 50-60 Gy delivered over 3-6 weeks (61). Currently, no chemotherapeutic treatments are involved in the SOC for pHGG; however, various are being tested in clinical trials (64). Despite immense efforts, there are no effective treatment options and pHGG has become the leading cause of cancer related death in children and adolescents under the age of 19 years (26, 60).

There is a diversity of molecular alterations driving pHGG and therapies must be accordingly diverse and specific. Highly targetable molecular alterations are found in different subtypes of non-brainstem pHGG. For example, pHGG often carry genetic alterations in the TP53, PTEN/PI3K/Akt, PDGF or Ras pathways, which include targets that can be druggable (65). However, immunotherapies specifically designed for pediatric brain tumors have been understudied. Pre-clinical models for pHGG and the testing of immune-mediated therapeutic approaches are starting to emerge (66, 67), which open new avenues for the treatment of these aggressive pediatric brain tumors.

## Glioma Immune Microenvironment

### Crosstalk Between the Healthy CNS and the Immune System

The brain has for long been considered an immune privileged site due to the absence of immune response after the heterotopic transplantation of skin xenografts (68). However, in the same set of experiments, Medawar et al. observed that if the immune system had been previously exposed to the tissue graft in any other site of the body and then the transplantation was done in the brain, a powerful immune response invaded the CNS, causing grafting breakdown and rejection (68). These data showed that the CNS in not immune-isolated and that even though an immune response against xenografts cannot be easily started in the brain parenchyma, it can reach this site in a pre-immunized state.

Due to anatomical particularities, the crosstalk between the CNS and the immune system differs from the immune response mounted in any other organ of the body (69–72). For instance, the passage of molecules and cells, such as immune cells, to the brain parenchyma is subjected to a strict control by the endothelial blood-brain barrier (BBB) (69). Also, the absence of classic lymphatic drainage in the CNS was considered to be the cause of the lack of an afferent arm of the immune system; i.e. the route of antigen transportation from the site of infection/trauma to the nearby lymphatic node (69). However, maintaining the brain as an immune-isolated tissue would be dangerous, thus many efforts had been destined to understand the mechanism by which the immune system surveils the CNS. There are two types of fluids in the CNS: the cerebrospinal fluid (CSF), in the ventricles and the subarachnoid space; and the interstitial fluid in the brain parenchyma. Even though both types of fluids drain to the cervical and lumbar lymphatic nodes, they do it through separate routes: while the CSF drains across the cribriform plate and the dura mater lymphatics, the interstitial fluid drains via perivascular channels into the lymph nodes or the CSF (69, 71). This narrow space does not allow the passage of cells, but it permits antigen transportation to the nearest lymph node, where adaptive immune response could be started. In contrast, the drainage pathways of the CSF allow cell trafficking and this fluid has a more active crosstalk with the immune system (69, 73). In fact, healthy individuals contain up to 700,000 cells in total in the CSF (70). Around 80-90 % of these cells are T cells, majority of which are memory T cells (70, 73). Also, a small proportion DCs has been found in the CNS, and there is evidence that DC can scan the CSF for foreign antigens and reach the lymphoid organs to activate T cells in the periphery (70, 72).

Even though these data demonstrate the interconnection between the immune system and the healthy CNS, this site usually remains quiescent and immunosuppressed due to the presence of factors derived from neural cells (70, 73). For instance, the brain parenchyma contains only one type of immune cell: the microglia. These cells are tissue resident macrophages, but they originate from a different embryonic layer than circulating macrophages (73, 74). These cells are kept in an inactivated state through the interaction of the CD200 receptor in neural cells and CD200 ligand in microglia (75). Even though these cells are capable of antigen presentation, the levels of MHC in microglia and other astrocytes remains low (73). However, in response to an infection, microglial cells become activated and produce an array of pro-inflammatory mediators, to facilitate the recruitment and activation of innate and adaptive immune cells (76). After an inflammatory stimulus, the immune privilege of the brain switches, increasing the permeability of the BBB and the infiltration of myeloid cells and activated T cells, as well as the proliferation of microglial cells (73, 76–78). This state causes phenotypic changes as well, such as CD11c, MHCII and co-stimulatory molecules’ upregulation (73, 78).

### Immune Microenvironment in Brain Tumors: General Concepts

The shift in the dogma of the CNS as an immune inert site, prompted the development of immunotherapies against glioma. Glioblastoma is one of the deadliest type of tumor and currently patients succumb to this disease even after their treatment with SOC (79). Thus, researchers have been devoted to find therapeutic alternatives to harness the immune system and direct it against this tumor. Today, there are several ongoing clinical trials testing different type of immunotherapies, but the results obtained so far have not been as encouraging as the effects observed in pre-clinical models and the great majority have not been tested in Phase III yet (12, 79).

There are several aspects related to the biology of gliomas that make them difficult to treat by immunotherapies. For instance, these tumors tend to have high intra-tumoral heterogeneity, so that finding a tumor specific antigen as a target for immune mediated therapies is difficult and usually approaches involving tumor antigens require the inclusion of more than one target to prevent antigen scape (80–82). Also, the intact BBB prevents the readily penetration of chemotherapeutics to the brain parenchyma, though its permeability can be affected in an inflammatory state (83). Finally, the immune microenvironment of these tumors tends to be immunosuppressive, hijacking the efficacy of immune mediated strategies (6, 8, 9, 12, 78).

Glioma tumor immune microenvironment (TME), refers to all those immune cells infiltrating the tumor mass. Even though the diversity of cell infiltration can vary depending on the type of brain tumor (revised below), glioma TME has usually been found to be immunosuppressive (6, 7, 11, 84). Animal models as well as the analysis of human samples have shed light on the characteristics of glioma TME. Myeloid cells are the major type of immune cell in glioma’s TME, with macrophages representing more than 30% of the tumor mass (6, 85). This group encompasses bone-marrow derived macrophages and tissue-resident derived macrophages (13, 74). It is not clear if these two populations have different functions in glioma or if they are associated with tumor progression, but they have been encountered at different locations: while microglial cells were found at the tumor border, bone-marrow derived macrophages were detected at the tumor core (86). These two types of cells are generally known as tumor-associated macrophages (TAMs). Also, infiltrating monocyte-derived macrophages constitute 85% of the total macrophage population in glioma and it has been observed that prevention of monocyte infiltration extended de median survival of tumor-bearing animals (86). There have been detected expression markers and differential transcriptional landscapes that can be used to distinguish these two populations (86–88). For instance, resident microglia express P2Y12, TMEM19, and are CD45 low, whereas macrophages express CD44, CD169 and are CD45 high (87). More importantly, these two cells’ subclasses have been identified in human samples, in which intratumoral blood-derived macrophages displayed a more immunosuppressive transcriptional program and their presence correlated with tumor malignancy (86).

Myeloid-derived suppressor cells (MDSC) are a type of immature myeloid cells that are known to have immunosuppressive functions via different mechanisms that ultimately inhibit T cell functions (9, 11, 13). These cells have been found in the blood of glioma patients and in the tumor mass, and they have also been characterized in animal models (9, 15, 89, 90). Usually, MDSCs are divided phenotypically in monocytic MDSCs (M-MDSCs) and polymorphonuclear MDSCs (PMN-MDSCs). In humans, M-MDSCs are characterized by CD11b+HLA-DR−CD14+CD15−CD33high, whereas PMN-MDSCs express CD11b+CD66b+CD15+CD14−/dimCD33dimHLA-DR− [PMCID: PMC6447515]. In mouse, MDSCs characterization entails less markers: M-MDSC are defined as CD45+/CD11b+Ly6G-Ly6C+ and PMN-MDSCs as Cd45+/CD11b+Ly6G+Ly6C- (91). It has been observed that the quantity and activation status of MDSC inversely correlates with patient survival and that they can be a predictor of WHO tumor grade (90). Moreover, whilst MDSC infiltration after surgery has been associated with poor prognosis, MDSC decrease correlated with better prognosis and an increase in DC infiltration (90).

Lastly, tissue hypoxia, which is common in GBM due to the inefficient neovascularization (10), induces regulatory T cells (Tregs) activation and tumor-promoting phenotype of tumor associated macrophages (10, 92). The presence of Tregs can suppress cytotoxic T cell activities, leading to tumor progression. Moreover, tumor cells as well as immunosuppressive tumor infiltrating immune cells, secrete an array of cytokines that promote and maintain the immunosuppressive microenvironment, not only affecting tumor infiltration, but also cellular differentiation at the bone marrow level (10, 84). Some of the cytokines encountered in the TME are IL-10, TGFβ and IL-6. These are related to NK and T-cell activities inhibition and their expression is related to glioma progression (93).

### Immune Infiltration Patterns in Brain Tumors With Different Genetic Landscapes: Lessons From The Clinic and Animal Models

It is clear that immunosuppression is a common feature of gliomas that enables tumor progression and malignancy. However, the composition of the immune cell infiltrate varies among the type of tumor and certain immune cells are associated with particular genetic alterations usually found in gliomas, such as mutations in *IDH1* (94).

The transcriptional landscape of GBM has been classified at least in three different types: proneural, classical and mesenchymal, which correlate with the presence of different genetic alterations (95, 96). This classification not only describes inter-tumor differences, but also intra-tumoral variability, as samples taken from distinct regions and at different times thought-out treatment showed diverse transcriptional signatures. With the emergence of Single cell RNA-Seq (scRNA-Seq), the cellular composition of glioblastoma was found to be even more complex. It has been observed that tumor cells can exist in four different phenotypes: mesenchymal-like, astrocyte-like, oligodendrocytic precursor cell-like and neural progenitor cell-like (80). These different cellular states are correlated with different genetic mutations and with the transcriptional signatures defined previously, with neural progenitor cell-like and oligodendrocytic precursor cell-like cells associated with the proneural subtype; mesenchymal-like cells with mesenchymal subtype; and mesenchymal subtype and astrocyte-like cells associated with classical subtype (80). This complexity in the phenotype of gliomas has been found to have a correlation with the composition of immune cell infiltrate (7).

Tumor microenvironment composition in adult glioma has been lately characterized. Luoto et al. performed a regression-based gene expression deconvolution to estimate the proportions of particular immune cell types based on RNA-Seq analysis of 156 primary GBM samples generated by The Cancer Genome Atlas (97). They found that cases could be grouped into three immune-response groups which were the following: negative, humoral and cellular-like. They also found that differences in adaptive immune response could be associated with the specific subtypes of HGG defined above. They describe that the “negative” subgroup, which is associated with the negative regulation of lymphocyte response, encompass the proneural subtype, including those samples with *CDK4-MARCH9* locus amplification and *IDH1* mutation. The mesenchymal subtype was more prevalent in the “humoral” subgroup, in which gene signature was related to B-cell and humoral response components. Finally, the “cellular-like” subgroup was more populated with classical subtype samples, as well as with samples with *EGFR* amplification. Also, they observed that immune-related responses correlated with the presence of specific genetic alterations. Samples with *CDK4* locus amplification or *IDH1* mutations were found to be less infiltrated by macrophages, and to have less CD4+ components. On the contrary, samples with *NF1* inactivation had a higher macrophage content. This observation has been confirmed in the study of Wang et al. (98). Even though none of the cell components described in the work by Luoto *et al.* correlated with patient survival, the presence of high activity related to the "antigen presentation and interferon response" cluster was a positive predictor of longer OS. Similarly, Caleb Rutledge W. et al., also found a correlation between tumor-infiltrating lymphocytes (TILs) and GBM transcriptional subclasses and they show that TILs were enriched in the mesenchymal class compared with all other classes (99). Also, they did not observe a correlation between *IDH1* mutation and TIL presence, nor did they with patient OS (99).

The correlation of *IDH1* status and TME composition has been extensively characterized (94). In general, as presented above, *IDH1*-mutant (mIDH1) gliomas tend to be less populated with TILs when compared to *IDH1*-wt tumors. Specifically, less CD8+ cytotoxic T cells have been found in mIDH1 gliomas and this could be explained by the reduced expression of chemoattractant cytokines to T cells by mIDH1 glioma cells (100, 101). Also, and in correlation to what Luoto *et al.* found, mIDH1 gliomas tend to have less macrophages than wt-IDH1 tumors (97). Moreover, in an animal model of mIDH1 glioma in the context of *ATRX* and *TP53* mutations, it has been observed that the presence of this mutation reprograms the tumor cell transcriptome, which affects not only immune cell infiltration but also the bone marrow differentiation of the granulocytic lineage (15). This effect was found to be mediated by G-CSF secretion by mIDH1 glioma cells, which prompted the expansion of pre-neutrophils, while reducing the immunosuppressive phenotype of the granulocytes encountered in mIDH1 tumors’ TME (15).

Tumor microenvironment in pediatric gliomas has been less characterized than the adult counterpart, in part because of the small amount of samples available. Thus, it is difficult to correlate molecular subtypes of pediatric tumors with TME infiltration patterns. However, the data gathered so far in the pediatric population show differences in relation to the immune infiltrate characteristics observed in adult patients. In the study of Plant et al., they analyzed 22 pediatric brain tumor tissue samples of mixed diagnoses and they observed no correlation between the amount of T cells and the aggressiveness of the tumor or the patient survival (102). Griesinger et al., analyzed different types of pediatric brain tumors, which consisted in 7 pilocytic astrocytomas (PA), 19 ependymomas (EPN), 5 GBM, 6 medulloblastomas (MED), and 5 non-tumor brain (NT) control samples. They show PA and EPN to be the most enriched tumors in myeloid cells, with GBM at the third place, but still with more myeloid cells than the NT samples (103). These cells expressed makers for both, immune activation (HLA-DR and CD64) and immunosuppression (CD206 and CD163). T cell infiltration was also evaluated and GBM had more T cells than the NT control (0.79% vs 0.02%), exhibiting a 46-fold and 26-fold increase in CD8 and CD4 T cells, respectively. Also, the average CD8/CD4 ratio, which was shown to be a prognostic factor in other types of cancer, was elevated in GBM with respect to NT controls: 2.83 vs 0.83, respectively (103). Moreover, Lieberman et al., studied the TME in DIPG, a pediatric high grade glioma that occurs in the pons. They conclude that the TME of these tumors do not show strong evidence of immunosuppression or inflammation, so that immune-directed therapies against these tumors should focus on immune cell recruitment to the tumor site (104). In this regard, Mendez et al. demonstrated the efficacy of an immunestimulatory gene therapy in increasing the median survival of tumor bearing mice in a pre-clinical mouse model for DIPG harboring mutant *ACVR1* gene (66). They show that this therapy was effective in promoting the activation and the infiltration of anti-tumor CD8 T cells (66). Lastly, using a model for pediatric HGG harboring the H3.3-G34R mutation it has been demonstrated that these tumor exhibit a more permissive TME with respect to the control group without the mutated histone (105, 106). Researchers show that H3.3-G34R tumors are less populated with MDSC and that these cells are not immunosuppressive. Also they observed an increased infiltration of T cells, DCs and M1 macrophages; and an increased sensitivity of glioma cells to IFNγ-induced apoptosis (105, 106).

In conclusion, these data gathered from clinical samples and pre-clinical models highlight the complexity of the immune cell infiltrate in brain tumors and the importance of taking into account the particularities of each type of glioma when considering the application of immune-mediated therapies.

## Mouse Models to Study Glioma Immune Microenvironment and Possible Therapies

The dismal prognosis of glioma patients demonstrates the need to faithfully model the formation and the biology of this tumor type to enable successful anti-glioma therapies. Immunotherapy has emerged as a promising approach to treat growing number of cancers (107, 108), but none has been effective in improving the survival of GBM patients (59, 109, 110). However, researchers working on GBM believe that immunotherapy could establish successful treatment regimens where other treatments have not been successful (111, 112).

Genetic, histological and physiological modifications are involved in the evolution of glioma’s malignancy and invasive phenotype. A good glioma animal model would enable the identification of signaling pathways which are related to tumor initiation, invasion, malignancy and therapeutic resistance. Ideally, the model should accurately resemble histologically and genetically the human disease. It should also display the cellular heterogeneity observed in glioma patients. Most glioma tumors have been previously modeled either in immunodeficient (113–115) or immunosuppressed (116) animals. However, these models have important drawbacks in terms of the lack of interactions with the adaptive immunity, which is key to fight this tumor. Also, tumors in immunocompetent mice exhibit characteristics similar to clinical pathophysiology in patients with glioma, characterized by immune infiltration and strong neovascularization, which are absent in brain tumors developed in immunodeficient mice (117).

Preclinical syngeneic murine glioma models are crucial to determine the immune response of novel therapies prior to its human clinical trial. The use of animal models of malignant glioma shed light on the composition of the TME, its influence on disease progression and outcomes, as well as on new therapeutic targets for treatment (118). The method widely used in glioma biomedical research is intracranial or subcutaneous injection of tumor cells like C6, 9L or GL261 into mice or rats. These syngeneic models are used to study the biology of glioma or new therapeutic agents. Also, there are other syngeneic murine glioma models, such as SMA560, GL26, CT-2A, 4C8 mouse models and 9L, RG-2, F98 and CNS-1 rat glioma models which maintain the immunological interaction between the tumor cells and the host (16).

GL261 model is perhaps the most extensively used syngeneic mouse model of GBM. This model is reported to recapitulate histologic and biological characteristics of GBM (16). Furthermore, this model employs immunocompetent mice, and thus is suitable to analyze GBM tumor immunology and to perform immunotherapeutic research (119). Among the reported pre-clinical applications of this model, we can mention: the use of adoptive T cell transfers to restore and induce long-term immunity; the use of antibodies to improve antitumor T cell activity via augmentation of costimulatory signals; the abrogation of survival advantages of Tregs; and the enhancement of tumor immunogenicity using IL12 based gene therapy to stimulate robust cytotoxic T cell responses (119), as GL261 express unique tumor antigens which can induce a specific T cell responses (120). Moreover, this model has been employed to study the immunosuppressive effects of TGFβ, which promotes Treg activity (121). Also, GL261 has been used to test the efficacy of a peptide vaccine using GL261-specific antigens and a TGFβ neutralizing antibody (1D11) (122). In another study, GL261-based DC vaccines have been curative and preventive of tumor engraftment (119). Thus, these results have helped to validate GL261 as one of the model of choice for investigating immunotherapeutic treatment modalities against GBM. Likewise, GL26 model enables the study of immunotherapies. GL26 tumors express melanoma associated antigens gp100 and tyrosinase-related protein 2, both of which can be used to pulse DCs, which would in turn stimulate cytotoxic T cell-mediated robust antitumor immune response (123). Other immune-mediated strategies tested with GL26 model include Treg depletion using PC61, which is an antibody directed against CD25, one of the primary markers for Tregs (124).

SMA-560 tumors are an excellent model of anaplastic astrocytoma with low S-100 expression and high expression of glial fibrillary acid protein (GFAP) and glutamine synthetase, providing a representative model of glial tumors of astrocytic lineage (125). These tumors lack MHC Class II molecules, but do express MHC Class I at low levels which highlights their potential for antigenic recognition by traditional effector T cells (126). They also express TGF-β which lends great value to this model (126). SMA-560 model has been used to test the efficacy of the induction of secretion of selected cytokines such as IL2, IL4, IL3, IL6 or TNFα, which resulted in an increase in MS of VM/Dk mice (126). Another study also showed that the over-expression of a soluble form of CD70 ligand in SMA-560 tumor cells, reduced tumor growth rate and increased host animal survival (127). Also, this model was used to investigate DC and CAR-T cell based therapies’ outcomes for radio-resistant glioma cells (128).

Histologically, CT-2A tumors show features of high-grade astrocytomas, including pleomorphism and high cellular density, and can undergo malignant transformation with evidence of pseudopalisading necrosis (129). Compared to established glioma cell lines, CT-2A cells are significantly more proliferative and invasive (130), but less invasive than other mouse brain tumors (131). CT-2A share similarities with neural stem cells, like primary human GBMs grown *ex-vivo*, and express stem cell markers such as CD133, Oct and Nestin (132). Overall, the CT-2A model is considered to accurately represent several GBM characteristics including intra-tumoral heterogeneity, *in vivo* migratory patterns, radio-resistance, and chemo-resistance (129). By virtue of its brain tumor stem cell-like properties, the CT-2A model could provide a resource for studying the role of tumor stem cells in the immunological landscape of gliomas. Moreover, since CT-2A is deficient in PTEN and this deficiency contributes to tumor induced immunosuppression (133), this model can be utilized to devise strategies for mitigating PTEN deficiency-associated immune effects (134).

4C8-B6D2F1 tumor model was developed to address the shortcomings observed with other glial tumors (135). The 4C8 cells adopt oligodendrocytic characteristics *in vitro*, but convert to GFAP+ astrocytes when exposed to serum (136). Implantation of 4C8 into B6D2F1 mice produces pleomorphic, highly cellular tumors with extensive invasion into ventricles and meninges (135). They also express components of MHC I and II molecules (137). Intratumoral injections with vaccines and viruses engineered to secrete IL-12, have shown to promote significant anti-tumor activity, with detected immune cell infiltration, and minimal toxicity (138).

The RCAS/*tv-a* system is a model that allows the somatic transfer of oncogenes driving glioma development, enabling the development of tumor *in situ*. This method has been used to initiate tumors in newborn mice, by the introduction of genetic alterations into brain cells engineered to express tv-a receptor (139). Genes used to initiate brain tumors could be *PDGF* and *Kras* overexpression. These animals can then be crossed onto other genetic backgrounds in order to study the effects of particular mutations on tumor biology (140, 141). It has been observed that the oncogenes *Kras* and *PDGF* produce more malignant gliomas in mice with *Ink4a-Arf-/-* and *PTEN* loss backgrounds compared with those gliomas generated in wt mice, which develop lower-grade tumors (139, 142). Hambardzumyan D *et al.* described a protocol to develop gliomas in adult mice, which represent an excellent tool for studying the tumor immune microenvironment and immunotherapeutic approaches in adult gliomas (141, 143). Even though this model has not been widely used for the study of glioma’s TME, it has been observed that, similar to what it is observed in the clinical setting, tumor malignancy of the gliomas generated with the RCAS system correlated with an influx of macrophages, which was influenced by tumor signal transducer and activator of transduction (STAT) 3 expression (144). In the same study, the authors report that STAT3 inhibition with WP1066 increased the MS of mice bearing brain tumors expressing PDGF-B + *Bcl-2* (144).

Another syngeneic model to generate gliomas *in situ* can be achieved by the Sleeping beauty (SB) transposon system (145). This method allows high-level stable gene transfer and sustained gene expression in many somatic cell types (146). The SB transposon system, member of the Tc1/mariner class of transposons, is capable of recognizing inverted repeats/direct repeats (IR/DR) sites on DNA transposons and performing a cut-and-paste reaction to integrate transposable DNA segments into a host genome (145, 147). This method has been used to develop endogenous tumors that mimic gliomas by delivering DNA transposons that encode for the genetic lesions of interest. Our laboratory has developed a series of syngeneic GBM models using this method. For instance, we have engineered ATRX-deficient gliomas (148, 149), by injecting plasmids encoding SB transposase/firefly luciferase, plus other plasmids encoding for the desired genetic alterations located between IR/DR: shp53, NRASG12V, and shATRX, into the lateral ventricle of neonatal mice (148). Also, using SB transposon system, we have developed: a DIPG murine tumor model of mACVR1-G328V by injecting plasmids encoding for NRASG12V, shp53, and mACVR1-G328V (66); a mIDH1 murine tumor model by injecting plasmids encoding for NRASG12V, shp53, shATRX and IDH1-R132H (44); and a H3.3-G34R murine high grade glioma model by injecting plasmids encoding for NRASG12V, shp53, shATRX and H3.3-G34R (67, 150). This method has the advantage that the tumors developed can be resected, processed as a single cell suspension, and grown *in vitro* as neurospheres. These neurospheres can be further implanted in adult C57BL/6 mice.

Recently, Patel SM et al. described a method for in utero electroporation of neural stem cells to generate an *in situ* mouse model for DIPG tumors, a highly aggressive glioma that grows in the pons in pediatric patients (151). They used PiggyBac DNA transposon plasmids to induce the expression of different combinations of PDGFB, *Pdgfra*-D842V, or *Pdgfra*-WT, along with dominant negative *Trp53* (DNp53) and H3.3K27M expression. They report the induction of gliomas from grades IV to II, which depended on the plasmid combination (151). These tumors displayed histopathological features of the human disease and represent an invaluable tool for the modelling of the TME in DIPG, as the development of the gliomas in this model resembles their development in humans. Also, to better depict the inter-person heterogeneity in immune response and glioma genetic make-up, Aslan K et al., described the use of an hypermutated orthotopic glioma syngeneic mouse model, exhibiting more than 100 non-synonymous mutations per tumor exome. This model was used to study the dichotomy in the glioma response to immune-checkpoint blockade and to develop a method to try to predict the therapy outcomes by imaging MRI technique (152).

An alternative humanized mouse model system has also been developed to evaluate the efficacy of various GBM immunotherapies. Humanized models are generated by the engraftment of human cancer cell lines, or human patient-derived xenograft (PDX) tumors into immunodeficient NSG mice with an HLA-matched human immune system, which is achieved by the transplantation of human PBMCs, or CD34+ hematopoietic stem cells (HSCs). Transplanted CD34+ HSCs in immunocompromised mice differentiate into human helper and cytotoxic T cells, B cells, monocytes, NK cells, and DCs (153). Humanized mice can survive months post-tumor implantation with relatively stable proportions of human cells. Human microglia/macrophage-like cells have also been developed in the brain of CD34+ HSC humanized mice (154). These models have the advantage of recapitulating tumor heterogeneity and clonal diversity, which mimics the human tumor immune microenvironment and can be used to investigate the biology of GBM (109, 155). Nevertheless, the humanized mouse platform is being improved in such a way that immunotherapeutic research could become more predictive. The use of humanized mouse models in GBM preclinical and clinical studies is currently limited due to the lack of knowledge and unanswered questions, such as whether humanized mice models display the clinical features of glioblastoma patients. For instance, Ashizawa T. *et al.*, investigated the efficacy of the anti-PD-1 antibody using humanized NOG-dKO mice, generated by implanting human PBMCs and GBM cell line U87 (156). In this study, there was no rejection of the human glioma cells or the PBMCs, and T-cell and NK-cell anti-tumor immune responses were detected, thus constituting an interesting model to evaluate the effect of immunotherapeutic agents against glioma. Despite these advantages, humanized mouse models are partial in maintaining the cellular and mutational diversity of parental tumors and entail an extended generation time (157, 158). Patient-derived glioblastoma organoids (GBOs) that recapitulate the histological features, cellular diversity, gene expression, and mutational profiles of their corresponding parental tumors have recently been developed and biobanked. When GBOs are transplanted into adult rodent brains, they show rapid, aggressive infiltration and high reliability (157).

While there is no perfect murine model to study immunotherapies for glioma, syngeneic tumor models in immunocompetent mice represent a valuable resource for this purpose. The transplantable models presented are convenient because tumor location and growth can be better predicted and thus, the testing of different therapies and their relationship with the immune system can be more easily studied. Although orthotopic xenografts retain some of the human GBM features and are considered to be a useful model for therapeutic studies (159), it lacks the proper immune environment due to the use of immunocompromised mice, which is a drawback for the study of tumor immunology and anti-tumor immune-stimulatory therapies.

## Strategies to Overcome Immunosuppressive Microenvironment: Current Therapeutic Modalities Under Clinical Trial and Under Pre-Clinical Investigation

### Immune Checkpoint Blockade

Immune checkpoints (IC) are negative regulators of the immune system that maintain self-tolerance, avoid autoimmunity and adjust the extension and duration of the immune responses to prevent tissue damages (160). The mechanism involves the interaction between IC receptors with its ligands, acting as a natural feedback loop that inhibits and reduces inflammation. Likewise, cancer cells could express IC ligands as a way to evade immune-mediated elimination. Examples of these immunomodulatory molecules, which are negative regulators of T cell activation and function, include the cytotoxic T lymphocyte antigen 4 (CTLA-4), the programmed cell death 1 (PD-1) and its ligand PD-L1, TIM-3, the enzyme indoleamine 2,3-dioxygenase (IDO), V-domain Ig suppressor of T cell activation (VISTA), killer-cell immunoglobulin-like receptor (KIR), TIGIT, B and T lymphocyte attenuator (BTLA) and LAG-3. Amongst them, CTLA-4, IDO and PD-1/PD-L1 are the most studied molecules inhibitors for which have been developed and evaluated in preclinical and clinical assays (160).

First attempts in developing IC inhibitors (ICIs) were focused in CTLA-4 molecule. CTLA-4 is a co-inhibitory receptor present on the surface of Treg that was discovered in the late 80´s (161). CTLA-4 has the B7 family of proteins (B7-1 or CD80 and B7-2 or CD86) as natural ligands, which are found at the surface of antigen presenting cells (APC). Even though CTLA-4 shares structural and biochemical similarities with CD28, a potent co-stimulatory receptor of T cells, CTLA-4 and CD28 have opposite immunoregulatory functions. Binding of CTLA-4 to B7 ligands has a 20-100 fold higher affinity than CD28, so when both are present, T cell activation is prevented and cytokine production switches to an immunosuppressive pattern, i.e. IL-10, TGFβ, and indoleamine (162).

Despite the lack of correlation between CTLA-4 ligand expression and a specific cancer cell type and the fact that *Ctla-4*-knockout mice models predicted lethal autoimmune phenotypes, it was shown that CTLA-4 inhibition produced antitumoral responses in preclinical cancer models (163). These preclinical studies showed promising results in some immunogenic tumors, using antibodies as a single agent or in combination with other agents that stimulated immune responses after tumor implantation, in the case of poorly immunogenic cancer models (163). Therefore, the development of fully humanized anti-CTLA-4 antibodies led to clinical testing of Ipilimumab and Tremelimumab. The first clinical study of CTLA-4 antibody treatment was performed in patients with advanced melanoma that were not responding to conventional therapy (164). Ipilimumab is a IgG1 monoclonal antibody that blocks the CTLA-4/CD80-CD86 interaction on APCs and T cells, promoting co-stimulatory binding of CD28 to CD80/CD86 (165). On the other hand, Tremelimumab is a monoclonal IgG2 antibody with a similar CTLA-4 blocking mechanism; but it only received orphan drug designation from the FDA for malignant mesothelioma (166).

Several pre-clinical trials evaluated the effects of CTLA-4 inhibition in GBM mouse models, showing differences in the outcomes depending on the tumor model evaluated. CTLA-4 blockade alone resulted in 80% of long survivors and abrogated Treg expansion in SMA-560 tumor-bearing mice (167). However, in other studies, the efficacy of this treatment was much lower, with 40 to 15% long term survivors (168, 169), or did not elicit antitumor efficacy (9). A significant challenge to effectively asses the efficacy of ICIs in GBM is to develop better pre-clinical animal models. In this sense, the SB28 GBM model recapitulate human GBM features, like low mutational levels and loss of MHC-I expression (170). Besides these technical issues, the sole inhibition of CTLA-4 in the immunologically suppressed microenvironment of GBM may not be effective to trigger a successful antitumoral immune response since this receptor is only present on T cells (171). In fact, our previous findings indicate that although the treatment with anti-CTLA-4 in GL26 GBM-bearing mice did not elicit antitumor effects, it boosted the efficacy of immune-stimulatory TK+Flt3L gene therapy (9). Moreover, even though preclinical data of CTLA-4 inhibition showed potential effects for GBM treatment, several adverse effects occur through a rapid and nonspecific activation of the immune system. In this regard, a Phase 1 clinical trial of Nivolumab (anti-PD-1) alone or in combination with Ipilimumab in patients with recurrent GBM showed no differences in OS but higher toxicity with the addition of anti-CTLA-4 to the treatment (172).

PD-1, which was first identified in 1992 as a putative pro-apoptotic receptor (173), plays a major role in limiting immune response and regulates T cell biology (174). While CTLA-4 acts early on T cell activation inhibition in the lymph nodes, PD-1 immune checkpoint controls the activity of T lymphocytes in peripheral tissues (175). PD1 ligand 1 (PD-L1; also known as B7-H1 and CD274) and PD-L2 (also known as B7-DC and CD273) serve as ligands for PD-1. They are present constitutively on resting T cells, dendritic cells, B cells, natural killer cells and macrophages, and can be induced in non-haematopoietic tissues by pro-inflammatory cytokines (176, 177). Specifically, tumor cells can express these ligands, protecting them from immune system eradication (178). For instance, the term “innate immune resistance” makes reference to *PDL1* gene amplification or the upregulation of PD-1 ligands by constitutively active signalling pathways on tumor cells (179–182). On the other hand, the “adaptive immune resistance” situation makes reference to PD-L1 expression by tumor cells in response to IFNγ release by T cells (183, 184).

The interaction PD-1/PD-L1 provokes effector T cells cell cycle arrest and the down-regulation of cell survival molecules like Bcl-XL, the dephosphorylation of ZAP70, and the phosphorylation of PI3K by the recruitment of SHP1 and SHP2 phosphatases (185). PD-1/PD-L1 axis disruption was thought to be a promising approach to overcome T cell inhibition and to promote an antitumoral immune response. In this regard, numerous studies have shown successful results in the treatment of metastatic melanoma (186, 187), Non-small cell lung cancer (188) and renal cell carcinoma (189). Preclinical studies using orthotopic mice models of GBM showed that PD-1 inhibition promoted NK cytotoxic effects against cancer cells when used as a single agent (190, 191) or in combination with radiotherapy (192). However, most clinical trial studies using anti-PD-1/PD-L1 monotherapy have shown limited efficacy in GBM patients (193).

Checkmate 143 was a Phase III clinical trial evaluating ICIs (Ipilimumab + Nivolumab) in GBM patients. It was concluded that Nivolumab as monotherapy was better than the combination, due to increased adverse effects when combined with Ipilimumab, and a significant increase in OS in comparison to the current therapy with Bevacizumab was not observed (194). In the case of PD-L1 inhibitors, a Phase 1 clinical trial of Atezolizumab as monotherapy in patients with recurrent GBM have shown no improvements in survival (195).

In conclusion, anti-PD-1 immunotherapy has been extensively evaluated in mouse models, and in clinical trials as monotherapy or in combination with other treatments, offering novel approaches for the treatment of GBM (**Table 1**) (12). Additionally, anti-PD-L1 immunotherapy has also been well evaluated in clinical trials (**Table 2**). Although the efficacy of ICIs as single agents has shown no satisfactory results in GBM, it is necessary to evaluate their efficacy as complements of other active immunotherapeutic strategies, such as vaccines and/or immune-stimulating gene therapies, which promote T cell infiltration, the subsequent IFNγ production and PD-1/PD-L1 upregulation.

**Table 1:** PD-1 inhibitor treatments approved by the FDA and in clinical testing for GBM patients.

Clinical trials investigating the use of ICIs for treatment of GBM					
Drug	Target	Name	Year	Clinical Phase	Arms
PD-1	Nivolumab	Neoantigen-based personalized Vaccine Combined with Immune Checkpoint Blockade Therapy in Patients with Newly Diagnosed, Unmethylated GBM	Actual Study Start Date: October 31, 2018 Actual Primary Completion Date: May 25, 2020 Estimated Study Completion Date: February 26, 2021	I	Arm A: NeoVax + Nivolumab (at progression) Arm B: NeoVax + Nivolumab (at Cycle 2) Arm C: NeoVax + Nivolumab (at Cycle 1)
		GMCI, Nivolumab, and Radiation Therapy in Treating Patients with Newly Diagnosed High-grade Gliomas	Actual Study Start Date: February 27, 2018 Estimated Primary Completion Date: February 28, 2021 Estimated Study Completion Date: February 28, 2021	I	Arm A: MGMT Unmethylated patients; AdV-TK injection into resection cavity, valaciclovir 14 days, radiation after 8 days, TMZ after valaciclovir, Nivolumab every 2 weeks to 52 weeks Arm B: MGMT Methylated and undetermined patients; AdV-TK injection into resection cavity, valaciclovir 14 days, radiation after 8 days, TMZ after valaciclovir, Nivolumab every 2 weeks to 52 weeks
		Translational Study of Nivolumab in Combination with Bevacizumab for Recurrent Glioblastoma	Actual Study Start Date: October 1, 2018 Estimated Primary Completion Date: February 1, 2022 Estimated Study Completion Date: August 1, 2022	II	Arm A: Nivolumab + Bevacizumab in patients not undergoing salvage surgery Arm B: Nivolumab + Bevacizumab in patients not undergoing salvage surgery
	Pembrolizumab	Combination Adenovirus + Pembrolizumab to Trigger Immune Virus Effects (CAPTIVE)	Study Start Date: June 2016 Estimated Primary Completion Date: December 2020 Estimated Study Completion Date: June 2021	II	Intratumoral DNX-2401 (a genetically modified oncolytic adenovirus) followed by IV Pembrolizumab
		Laser Interstitial Thermotherapy (LTTI) Combined with Checkpoint Inhibitor for Recurrent GBM	Actual Study Start Date: November 29, 2017 Estimated Primary Completion Date: December 2020 Estimated Study Completion Date: January 2021	l/ll	Arm A: IV Pembrolizumab 7 days pre-surgery with LITT Arm B: IV Pembrolizumab 14 days post-surgery with LITT Arm C: IV Pembrolizumab 35 days post-surgery with LITT
		PVSRIPO and Pembrolizumab in Patients With Recurrent Glioblastoma	Estimated Study Start Date: September 2020 Estimated Primary Completion Date: March 2023 Estimated Study Completion Date: March 2023	I	Single Arm: PVSRIPO intratumoral infusion followed by intravenous Pembrolizumab 14 to 28 days later, and every 3 weeks, thereafter

**Table 2:** PD-L1 inhibitor treatments approved by the FDA and in clinical testing for GBM patients.

Clinical trials investigating the use of ICIs for treatment of GBM						
Target	Drug	Clinical trial ID	Name	Year	Clinical Phase	Arms
PD-L1	Avelumab	NCT03047473	Avelumab in Patients with Newly Diagnosed Glioblastoma Multiforme	Actual Study Start Date: March 10, 2017 Estimated Primary Completion Date: September 2022 Estimated Study Completion Date: September 2022	II	Addition of Avelumab to standard therapy of TMZ and radiotherapy

IDO is an enzyme with an essential role in the catabolism of tryptophan (Trp) into different metabolites, like kynurenines (Kyn). Although it is not considered as a classical checkpoint, it is included in this group of molecules because it has powerful immunosuppressive properties (196, 197). IDO expression in the context of tumor immunity has been associated to cancer and immune cells (198). IDO contributes to immunosuppression activities by increasing Kyn levels and depleting Trp, which inhibit effector T cells and NK cells, and promotes Treg proliferation (198). This enzyme has been shown to be upregulated in almost all GBM patients (199) and its high expression correlates with malignancy (200). In this sense, a pre-clinical study of TMZ in combination with an IDO inhibitor showed tumor growth reduction and an increase in long-term survival of mice with GBM (201). Encouraging preclinical results led to several clinical trials with IDO1 inhibitors, but unfortunately administration as single agent did not show significant antitumoral activity. Nowadays, several clinical trials are being conducted in order to test IDO inhibition efficacy, in combination with TMZ and radiotherapy (NCT03532295, NCT02502708 and NCT04049669). Similarly, another trial tested the combination of an IDO inhibitor (INCB024360) with Nivolumab, Anti-GITR MAb and Ipilimumab in patients with recurrent GBM (NCT03707457). However, after a failed Phase III trial in melanoma, with no differences in progression free survival (PFS) or OS, it was proposed that IDO is not an appropriated target in cancer (202). However, it is possible that more effective and specific inhibitors need to be developed in order to successfully block IDO pathway in cancer (203).

Trp degradation to Kyn by IDO1 and TDO2 provokes Trp starvation, which causes the subsequent activation of general control nonderepressible 2 (GCN2), decreasing general protein production. IDO-activated GCN2 also affects T cells proliferation and effector function, by inhibiting fatty acid synthesis, promoting T-regs activation (204), Platten, 2012 #122}. In this sense, Trp degradation has been recognized as an important microenvironmental factor with immunosuppressive properties. Particularly, the IDO/TDO-Kyn-AhR enzymatic cascade has emerged as an interesting pathway to develop novel therapeutic strategies and overcome tumour immune scape in GBM. In this regard, besides several IDO inhibitors that are being tested in preclinical and clinical trials, Kyn has been shown to be an interesting target due to its aryl hydrocarbon receptor (AhR) agonist activity. AhR activation promotes the generation of immune-tolerant DCs and T-regs (205). Thus, the approach of depleting extracellular Kyn has shown promising efficacy in mouse models. Engineered KYNase catalyses the synthesis of anthranilic acid from Kyn, promoting effector T cell infiltration into the tumour (206). Finally, several AhR antagonists are being tested in preclinical studies (207). However, due to their broadly effects inhibiting any AhR ligand (endogenous and exogenous), development of antitumoral AhR therapies is in early stages (207). In this sense, it remains to be elucidated if this approach will show anticancer activity by acting on cancer cells or by modulating immune responses, and if they achieve optimal pharmacokinetic/pharmacodynamic profiles (204).

### Macrophage Reprogramming

The heterogeneous microenvironment of glioblastomas contains an enriched proportion of non-tumor cells which characterize the TME. Although the quantity of lymphocytes is very low, the tumor-associated macrophages (TAM) have been described as one of the major populations of GBM’s TME. TAM comprises two main subpopulations, the microglia (MG) and the monocyte-derived macrophages (MDM) (208, 209). MGs are the resident immune cells of the central nervous system specialized to monitor and respond to pathogens or injuries (210) and MDMs are peripheral bone marrow derived cells that infiltrate the TME. Despite different origins, these two populations function as immune-suppressed cells of the TME which diminish T-cell response and promote tumor progression and invasion (209). Different studies have shown that the pro-tumorigenic role of TAM is promoted by their interaction with glioma cells through mutual paracrine signaling. Different released factors have been involved in TAM-GBM interaction and the shift of TAMs to an M2 phenotype, which is characterized by anti-inflammatory properties and reduced phagocytic activity. Several studies have demonstrated that CSF1 could act as MG chemoattractant and that TAM released factors, such as the epidermal growth factor (EGF), TGFB1, IL-10, TNF, MMP14, MMP2, can promote GBM migration and invasion (211–213). Due to the immune-suppressing role of TAM in tumor progression, this population became a novel target for antitumor immunotherapies (213).

**Figure 1:**
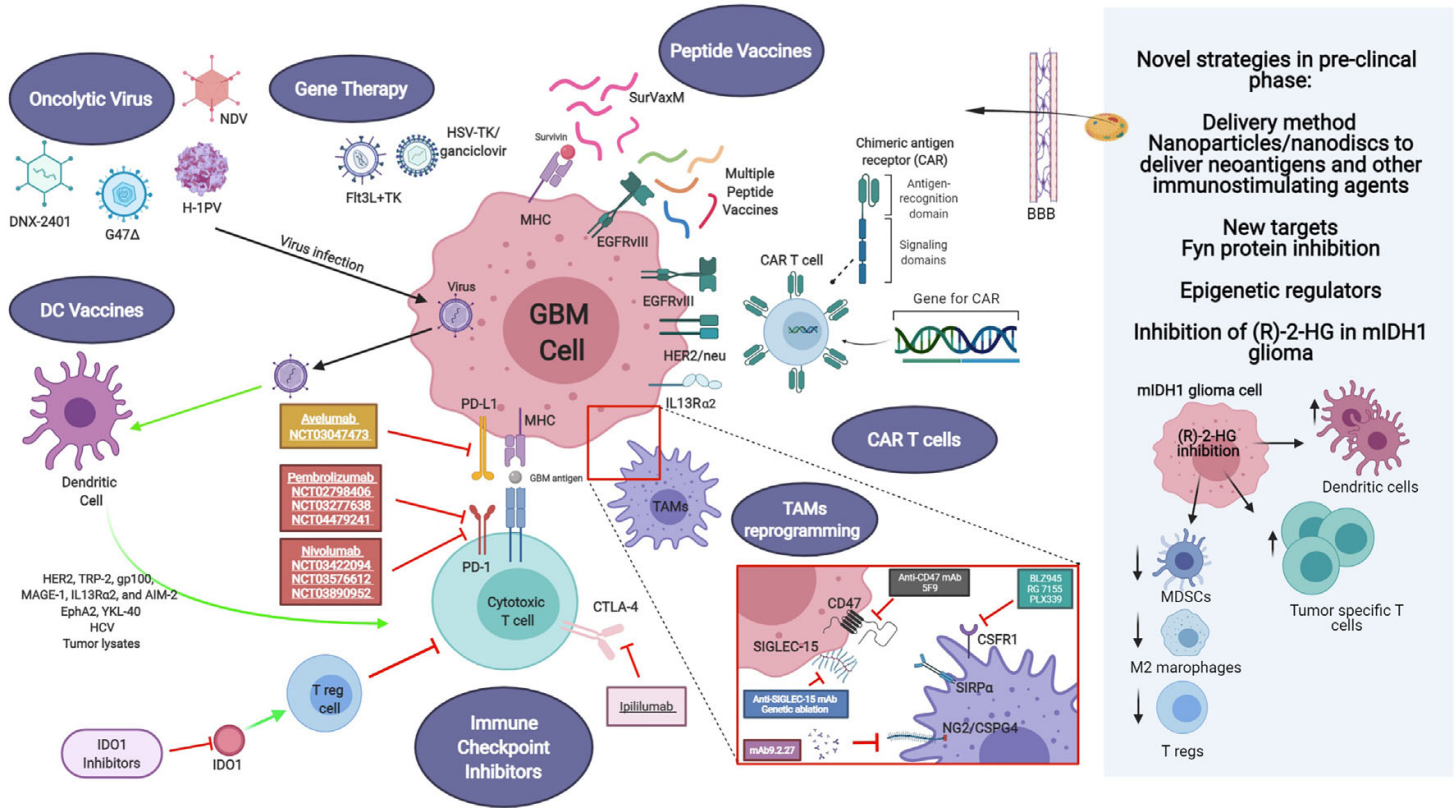
Current and novel immunotherapeutic strategies for GBM treatment under pre-clinical and clinical investigation. Current immunotherapeutic strategies in GBM include oncolytic viruses that can destroy glioma cells through immunogenic cell death without affecting non neoplastic brain cells, TAM reprogramming and the use of CAR T cells. Activation of immune checkpoint ligands such as PD-1, CTLA-4, and IDO can help tumor cells to escape immune surveillance. Thus, inhibition of them can effectively inhibit glioma progression and improve the response to other active immunotherapeutic strategies, such as DC vaccines and immunostimulant gene therapy. GBM antigens, including IL-13Rα2, HER2/neu and EGFRvIII are present in tumor cells. These tumor-associated antigens are targets of genetically modified CAR-T cells or peptide vaccines. Also, novel strategies are being studied currently in the pre-clinical setting, addressing more efficient ways to cross the blood-brain barrier (BBB), such as nanodiscs, and the modulation of the activity of novel targets. Created with BioRender.com.

Different strategies have been focused on the impairment of macrophage tumor recruitment and on the reprogramming of phagocyte innate immune surveillance functions of MDM and MG (**Figure 1**).

Recent studies have shown that the CSF1 ligand is expressed in glioma cells and TAM. The CSFR1R is only expressed on macrophages (214). Inhibition of CSF1R using the blood-brain barrier permeable compound BLZ945, significantly decreased tumor growth and extended survival in a mouse model of GBM and patient-derived xenografts models. Treatment efficacy was related to M2 macrophage polarization inhibition, but not TAM depletion in tumor treated mice. Molecular analysis of TAM showed that this population had an inhibited expression of some M2 polarization markers, such as Arg1, F13a1, Mrc1, and Adm (215). Although, different inhibitors such as BLZ945, RG 7155, PLX339 have been tested in clinical trials, blocking of CSFR1 remains challenging and requires further studies.

TAM survival is maintained by factors released by glioma cells, such as interferon-γ (IFN-γ) and granulocyte-macrophage colony stimulating factor (215, 216).

The combined treatments of CSFR1 inhibitors with PD-1 or PDL-1 monoclonal antibody are promising avenues under investigation in clinical trials (216). Also, the combination of triple therapy using checkpoint inhibitors (anti-CTLA-4 and anti-PD-1), and immune-virotherapy showed effective M1 polarization of macrophages and tumor eradication (217). Other combinational therapy approach inhibiting the Neuroglial-2/Chondroitin sulfate proteoglycan-4 (NG2/CSPG4) axes using the antibody mAb9.2.27 together with activated NK cells in preclinical animal models of gliomas decreased tumor growth, by increasing recruitment of CCR2low MDM to the TME and by amplifying ED1 and MHCII expression on MG (218).

Likewise, other studies showed that therapies using dual inhibitors of VEGF and Angiopoietin-2 (ANG-2) axes led to modifications in TAMs. A2V bi-specific antibody or dual therapies utilizing Cediranib and MEDI3617, reprogramed macrophages to antitumor M1 phenotype, inhibited TAM recruitment and delayed tumor growth and progression (219, 220).

Further research studies demonstrated that CXCR4 signaling is involved in the recruitment of TAM to the TME. Inhibition of this axis using the clinically approved drug AMD3100 prevents BMDCs infiltration, tumor revascularization, and abrogate tumor recurrence (218). Moreover, a recent phase I/II clinical trial study showed positive results in macrophage recruitment inhibition and local control of tumor recurrences after irradiation therapy using a reversible CXCR4 inhibitor Plerixafor (221).

Glioma cells redirect macrophages activating signals to a pro-inflammatory M2 state. This strategy has been related to the overexpression of Spp1 (secreted phosphoprotein 1 or osteopontin) and Mgfe8 (milk fat globule-EGF factor 8 or lactadherin) on glioma cells and human glioblastoma tissue, which prompt M2 reprogramming of MG as a result of integrin signaling activation. Furthermore, downregulation of Spp1 and Mgfe8 within glioma cells inhibits the amoeboid transformation of myeloid cells and redirect M2 microglia/macrophages phenotype impairing glioma growth (222).

The latest strategies developed have been related to the targeting of CD47/SIRPA (signal regulatory protein alpha) pathway. CD47 is a transmembrane protein overexpressed in glioma cells that binds to the receptor SIRPA on the surface of monocytes/macrophages and MG cells inhibiting phagocytic functions and allowing tumors to escape the innate immune surveillance. Transcriptomic analysis of human gliomas has shown that high expression of CD47 correlates with overall survival, which makes CD47 a novel prognostic marker (223, 224). The mechanism of activation of CD47/SIRPA includes the activation of ITIM (immune-receptor tyrosine-based inhibitory motif) and subsequent signalling through the activation of PTPN6 (protein-tyrosine phosphatase non-receptor type 6) and PTPN11, inhibiting phagocytosis (225). Moreover, preclinical studies based on orthotopic glioma models showed that blocking CD47 using antibodies decreased tumor growth and enhanced animal survival (223). Even though the major role of CD47 inhibition has been attributed to peripheral macrophage recruitment, Hutter, G. et al also demonstrated its effect on resident microglia. Using mouse glioma models which enable the differentiation of genetically labelled MDM (Ccr2 RFP) and MG (Cx3cr1 GFP), they showed that microglia associated tumor cells increase tumor cell phagocytosis in response to CD47/SIRPA axis inhibition (226). This data indicates that enhancement of MDM and MG phagocytosis phenotype is a promising avenue for glioma treatment. Recent clinical trial studies using 5F9, a CD47 inhibitor, on other solid tumors showed a positive response in combination with other anticancer treatments (227).

Another feature of malignant transformation in glioma is the protein over-glycosylation ended by charged sialic acid in glioma cells, which constitutes novel target. SIGLEC (sialic acid-binding immunoglobulin-like lectin) proteins (14 different identified variants) are receptors of sialic acid and they are mainly present on immune cells (TAMs) acting as negative regulators of phagocytosis. SIGLEC receptor activates immunosuppressive signals after binding to sialic acids through the same signalling pathways activated in the CD47/SIRPA axe as discussed above (213, 228, 229). Examination of the sialic acid/SIGLEC pathway has demonstrated that genetic and antibody ablation of SIGLEC15 expands anti-tumor immune response and obstructs tumor growth in mouse glioma models (230).

In summary, these studies show that regulation of TAM recruitment to the tumor mass or re-education to a phagocyte phenotype contributes to the anti-tumor response and inhibition of glioma progression. Due to the diversity and plasticity of TAMs, a better understanding of the mechanisms involved in TAMs recruitment and reprogramming remain a challenge to target these immune modulators of the TME for treatment. Combination of conventional therapies, immune checkpoint inhibitors together with TAMs regulation appears to be a promising alternative to improve glioma immunotherapy and halt glioma progression.

### Therapies Aiming at the Stimulation of the Immune System to Develop Anti-Tumor Specific Immune Response

An additional group of immunotherapies are aimed at inducing the development of antitumor specific responses, i.e. mediated by specific T-cells or antibodies production. These therapies were discussed in detailed before, and it is not the purpose of this manuscript to review them on detail. Anti-tumor specific response-inducing therapies can be summarized in:

- Oncolytic virus-mediated therapies, where oncolytic viruses are targeted to the tumor cells to cause Immunogenic cell death (ICD), stimulating the release of tumor-associated antigens (TAA) and damage-associated molecular patterns (DAMPs), which help to overcome the immunosuppressive tumor microenvironment (231). In this way, ICD induces the recognition of tumor cells by the immune system and the development of long-term immunity (232). Furthermore, OVs induce antiviral innate immune responses triggered by pathogen-associated molecular patterns (PAMPs) (231). Additionally, OVs can be genetically engineered to deliver immunotherapeutic transgenes or to increase their tumor selectivity, enhancing their potential for oncolytic immunotherapy (231–246).
- Suicide gene therapies, which comprises the delivery of genes encoding a conditionally cytotoxic enzyme that converts a non-toxic prodrug into a cytotoxic compound. In this way, transduced tumor cells are destroyed, sparing normal cells (247). The most evaluated suicide gene therapy for the treatment of GBM is HSV- thymidine kinase (TK) plus systemic administration of ganciclovir (GCV) (247).
- Peptide vaccines, where the main objective is to inhibit cancer progression or relapse, by producing humoral (tumor-specific antibodies) or cellular (cytotoxic T cells activation) responses against tumors (20, 248).
- Dendritic cells (DC) vaccines. DCs are professional antigen presenting cells (APCs), which function is to recognize, process and present antigens to T cells in the context of the major histocompatibility complex (MHC) I and II, in order to activate T cells and subsequently the adaptive immune response (12). Moreover, DCs are able to secrete pro and anti-inflammatory cytokines that modulate the tumor microenvironment. Autologous DCs can be loaded *ex vivo* with tumor antigens, peptides, tumor lysates, viral antigens, GSC or mRNA, among others, and then be administered back to patients as an antitumor vaccine (8, 249). These autologous DCs are usually differentiated from autologous monocytes by the incubation with specific cytokines (12).
- CAR T therapy. Chimeric antigen receptors (CARs) are recombinant receptors for specific targets found on cancer cells. They are designed to redirect the specificity and function of patient-derived cytotoxic T cells, which are *ex vivo* genetically engineered to express the CAR and re-infused to the patient (186, 250, 251).

## Influence of Genetic Alterations Present in Glioma Subtypes on the Response to Immunotherapies

Although the use of immune therapies to treat gliomas is still in its early stages, *i.e.*, in research and trial phases, the knowledge accumulated in the field in CNS tumors and other solid cancers indicate that the genetic makeup of the tumor is a predictive factor of the efficiency of the immune therapies. Not only the genetic information can predict if a treatment is likely to generate response or not, but also the genetic alterations present in certain subtypes of tumors can be exploited to devise tailored immunotherapies. In solid tumors, it was observed that the mutational load is a positive predictive factor of response to immunotherapy. A significant proportion of gliomas have mutations associated with DNA repair defects and genetic instability (252). As a consequence of this, higher mutational burden, has been observed, particularly in pediatric HGG patients with DNA repair-related germline mutations (252). Although a recent study found no correlation between mutational load and response to immune checkpoint inhibition in glioma (253), it is likely that the treatment in this study was inefficient due to the poor penetrance of the immune checkpoint inhibitors to the brain (254). In this respect, a patient with Lynch syndrome (a genetic condition related with mismatch DNA repair deficiency) who developed an IDH-mutant glioblastoma was treated with a PD-1 inhibitor (Nivolumab), remaining free of recurrence for 5 years (255).

Recent studies have described the peculiarities of the immune compartment in gliomas, which is strongly immunosuppressive and enriched in myeloid suppressor cells and exhausted and regulatory T-cells (209). Recently, it was demonstrated that the interactions between tumor cells and the immune microenvironment are influenced by the genetic alterations of the tumors. For instance, some studies reported that IDH mutations induce epigenetic changes that lead to establishing an immunosuppressive TME (94, 100). However, work from our team reported that infiltrating immune myeloid cells in the mutant-IDH TME are devoid of immunosuppressive properties. This highlights that it is not only important to identify the presence of a particular immune cells population using defined molecular markers, but also to assess the functional activity of these cells, to be able to describe the characteristics of the TME (15). In addition, it has been reported that mutant-IDH glioma cells express lower PD-L1 levels due to epigenetic reprogramming, suggesting a less immunosuppressive environment. All this information is pivotal to devise therapeutic approaches, *e.g.*, the concept of combining mutant-IDH inhibitors (to revert the suppressive TME) with immunotherapies. However, considerable work still needs to be done in regards to the genetic and functional characterization of immune cell populations in the TME of gliomas. Single-cell RNA-seq allows the identification of different immune cells within the glioma TME, and can inform on whether certain molecular subtypes of glioma have more immune-active or immune-suppressive environments. Another type of technologies that can shed light into the characteristics of the immune populations within glioma TME are the studies performed in *de novo* genetically engineered animal models, which can be developed in immunocompetent mice and allow to dissect the effect of particular genetic alterations in the immune TME (15, 44, 148). This type of studies will also help devising tailored immunotherapies for specific gliomas. For instance, a group of NF1-mutant low grade gliomas was demonstrated to be associated with immune activation, increased cytolytic T-cell infiltration and neoantigens production, and this group might benefit from immunotherapies (256).

Another area where immunotherapies benefit from the knowledge of the glioma genetic makeup is on the development of CAR-T therapies or peptide vaccines. CAR-T therapies require the identification of targets that are expressed on the surface of the tumor cells and that are not expressed in normal cells. Interleukin-13 receptor α2 (IL-13Rα2) was identified as a glioma specific marker, and CAR-T therapy with cells targeting this protein was evaluated in a clinical trial (257). The preliminary results of this study on a single patient reported glioma regression, but development of therapy resistance associated to the emergence of (IL-13Rα2) negative cells. Additionally, histone K27M-mutant cells show consistent expression of GD2, and its CAR-T-mediated targeting was efficient in K27M xerograph models (258). These studies provide the foundation on future directions to develop efficient immunotherapies for glioma. CAR-T cell therapies. The identification of multiple cell surface markers with minimal off-target effects for the different molecular subtypes of gliomas is essential to target tumors and prevent antigen escape-associated resistance. In this regard, the recent genomic analysis of gliomas has led to the identification of clonal mutations that drive the different molecular subtypes. For example, IDH1/2 mutations in adult gliomas and histone H3.1 and H3.3 mutations in pediatric high grade gliomas were shown to be clonal for their respective subtypes, and developing therapies targeting these genetic alterations would reduce the risk of antigen-escape.

The epigenetic alterations induced by driver mutations such as those in IDH1/2 and H3.1 and H3.3 histones may also induce DNA repair deficiencies and/or genetic instability, and this can be associated with more immune reactive tumors. For example, cells more susceptible to DNA damage, such as H3.3-G34R mutant cells (259), undergo immunogenic cell death (ICD) upon DNA damaging conditions, which can revert the immune-suppressive TME. For this reason, acknowledging the susceptibility of the different glioma molecular subtypes to different treatments to induce ICD. For example, HDAC inhibitors were shown to target K27M HGG (260), and other tailored therapies are being explored for other subtypes (252), but the potential combinations of treatments inducing ICD and immunotherapies remain unexplored.

In summary, it is clear that the genetic alterations present in the different glioma molecular subtypes are determinant of the efficacy of immunotherapies possibilities and responses, and that the evolving information of each glioma subtype will provide opportunities for novel tailored immunotherapies.

## Novel Targets and Strategies to Stimulate Anti-Glioma Immune Response

In this section, we aim to discuss about the latest glioma targets and anti-tumor strategies being studied in the pre-clinical setting (**Figure 1**).

### Fyn Inhibition as a Target to Enhance Immune Response and Prevent Tumor Progression

Despite current advances in the molecular characterization of gliomas and novel therapies to target the tumor immune microenvironment, treatment of glioblastoma remains elusive (34, 261). Latest studies indicate that glioma infiltrating myeloid cells inhibit the anti-glioma immunity and enhance tumor progression and thus, the identification of the connections between tumor cells and the tumor immune suppressive microenvironment could open innovative treatment options (9, 262). Fyn, a non-receptor tyrosine kinase member of the Src family kinases (SFK), has recently emerged as a novel regulator of the tumor immune microenvironment during glioma development (20, 248). Fyn regulates several cellular functions in normal physiology and is deregulated in different cancers (263–265). It has been shown that Fyn displays important functions related to the immune system modulation, regulating the activity of T cells (266, 267); and in the development of the CNS, regulating the migration and adhesion of neurons (268, 269). Previous studies on Fyn’s role in cancer, including glioma, show that Fyn is activated via NRAS dependent and independent pathways through the oncogenic receptors EGFR, PDGFR, HGF/MET or RTK/RAS/PI3K to increase cell migration, proliferation and reduce cell death (270–272). These growth factor receptors are the most common mutated driver genes in GBM tumorigenesis (34, 261). Even though Fyn is mutated in a very low percentage of human gliomas (0.1-0.4 %), it has been shown that it is overexpressed in higher grade mouse and human gliomas (20, 270).

Until recently, Fyn has been best known by its cell-autonomous functions. The majority of *in vitro* studies showed that pharmacological inhibition and genetic downregulation of Fyn in glioma cells decreased cell proliferation and migration (265, 270, 273, 274). However, *in vivo* studies had been inconclusive (275). Recently, Comba et al. demonstrated not only the effects of Fyn in increasing glioma cell proliferation and migration, but also an unusual cell-non-autonomous role of Fyn inhibiting the anti-glioma immune response (20). RNA-Seq and network bioinformatic analysis of the tumor transcriptomic landscape on glioma mouse model tumors indicated that Fyn’s biological effects were related to the immune microenvironment. Using diverse genetically engineered immune-competent mouse glioma models, the study shows that genetic downregulation of Fyn increases survival and decreases glioma growth and progression. Interestingly, Fyn knockdown tumors generated in immune deficient mice (NSG, CD4-/- and CD8-/-) exhibited no differential effects on tumor growth and survival, demonstrating the relevance of the immune response in the progression of these tumors. The mechanistic analysis showed that Fyn depletion reduces the expansion of the immune suppressive myeloid cells (MDSCs) in the TME, including monocytic-MDSCs (CD45+, CD11b+, Ly6Chi, Ly6G−) or polimorphonuclear-MDSCs (CD45+, CD11b+, Ly6Clo, Ly6G+) and therefore less inhibition of T-cell activity is observed. Fyn increases tumor growth due to MDSC migration induction, their augmented expression of ARG1 and CD80, as well as their enhanced functional immunosuppressive activity (20). This work opens up new avenues for future investigations to understand glioma-immune microenvironment cross-talk and increases the potential efficacy of anti-glioma therapeutics. All these data suggest that Fyn inhibition in tumor cells is a novel therapeutic target for glioma treatment. Inhibiting Fyn’s pro-tumoral activity has the combined effects of reducing tumor cell proliferation and migration, as well as inducing the anti-tumor immune response.

The incapacity of different therapeutic agents to cross the blood-brain barrier and the non-specificity of the available Fyn pharmacological inhibitors challenge the possibilities of its use as a target for glioma treatment (265). To investigate the translational implications of targeting Fyn in glioma, we propose the use of glioma pre-clinical models to test the efficacy of nanoparticles loaded with small interfering RNA (siRNA) against *Fyn*. This strategy has the advantage of being systemically administrated, since nanoparticles have the ability to cross the brain-barrier and deliver their cargo directly into brain tumors (276, 277). Furthermore, the combination of Fyn inhibition within glioma cells and cancer immunotherapy, such as immune checkpoint blockade (PDL1 and PD1 inhibitors), IFNγ therapy, and Ad-hCMV-TK plus Ad-hCMV-Flt3L immune-stimulatory gene therapy (8, 12, 278), are promising avenues to improve the efficacy of anti-glioma immunotherapies and explore novel personalized treatment for glioma patients.

### Nanoparticles as a Novel Anti-Glioma Therapy to Stimulate the Immune System

Nanotechnology is a potentially promising strategy to utilize against gliomas. It offers advantages such as (1) targeted delivery of materials to specific organs and tissues; (2) antigen and adjuvant co-delivery to antigen-presenting cells (APCs); and (3) non-invasive delivery of therapeutics; while (4) providing safe and biocompatible platforms for combinational immunotherapy, especially with immune checkpoint blockade (ICB) (279). In particular, Kuai, et al. have developed a synthetic high-density lipoprotein (sHDL) nanodisc platform composed of phospholipids and apolipoprotein A1-mimetic peptides (280). As a platform for cancer immunotherapy, sHDL has ideal properties, including multiple cargo loading sites for antigens, adjuvants, and chemotherapeutics, and its small size (10 nm) mediates efficient delivery of cargo to draining lymph nodes or directly to tumors for cytotoxic effects. In this regard, we have demonstrated strong anti-tumor efficacy of sHDL delivering GBM neoantigens or a chemotherapeutic agent docetaxel (DTX) in murine models of glioma (21, 22).

The sHDL nanodiscs using GBM neoantigens were synthesized by modifying neoantigen peptides with a reduction-sensitive cysteine-serine-serine linker, which was reacted with a dioleoyl-*sn*-glycero-3-phosphoethanolamine-*N*-[3-(2-pyridyldithio) propionate] (PDP)-modified lipid to produce neoantigen-lipid conjugates (22, 280). Loading of neoantigen-lipid conjugates and CpG (a Toll-like receptor-9 agonist) in sHDL was mediated via hydrophobic interactions after simple mixing. Nanodiscs were taken up by DCs, leading to strong localization with endosomes/lysosomes, sustained epitope-MHC I presentation, and cross-priming of CD8+ T cells against GBM. Mice were inoculated with an orthotopic GL261 model and treated with a combination of nanodiscs carrying three GBM neoantigens and anti-PD-L1. The results showed up to 100-fold higher IFN*γ*+ T cell responses and eradicated 30% of gliomas, compared with soluble vaccine + anti-PD-L1 treatment (22). Furthermore, there were no signs of recurrence through day 90, on which mice were re-challenged in the contralateral hemisphere and did not show any signs of neurological deficit as well (22). These results are particularly exciting as they demonstrate immunological memory and the ability of glioma-specific T cells to traverse the blood brain barrier (BBB) and exert cytotoxic effects against gliomas.

Nanodiscs carrying DTX and CpG were synthesized similarly (21). One of the main barriers of effective glioma treatment is the BBB, which provides a physical resistance to GBM chemotherapeutic treatment. By loading sHDL nanodiscs with DTX and CpG and injecting them intrathecally, the BBB was bypassed, allowing the nanodiscs to diffuse through the entire tumor (21, 281). When sHDL-DTX-CpG was administered to orthotopic GL26 tumor-bearing mice, a ~2-fold increase in survival was observed, compared with DTX, DTX-CpG, or DTX-sHDL treatment (21). sHDL-DTX-CpG triggered immunogenic cell death, as evidenced by high expression of “eat me” and “danger” signals, such as calreticulin and HMGB1 on the surface of tumor cells. sHDL-DTX-CpG also promoted recruitment of APCs as well as CD8+ T cells into GBM tumors (21). As standard therapy for GBM is normally a combination of radiation therapy and chemotherapy, GBM-bearing mice were treated with a combination of sHDL-DTX-CpG and radiation therapy, which resulted in 80% tumor regression and no tumor recurrence post tumor re-challenge (21). Exemplified by these two examples using the sHDL nanodisc platform, nanotechnology is a novel and effective therapy to stimulate a comprehensive anti-GBM immune response.

### Current Therapies Aimed at Targeting Epigenetic Pathways in Glioma and Its Impact on the Immune Response

Insights into the molecular landscape of diffuse gliomas have revealed characteristic genetic and epigenetic profiles which stratified the glioma classification (38, 47). Genetic anomalies associated with gliomagenesis commonly coincide with specific epigenetic mutations (282). These include but are not limited to a mutation in histone H3 genes such as H3K27M, and H3G34R/V as well as a mutation in the epigenetic modulator gene isocitrate dehydrogenase (IDH) (38, 47). Owing to the reversibility of epigenetic modifications, the proteins and genes that regulate these changes have become new targets in the treatment of glioma (282). Epigenetic mechanisms are critical for many processes in cancer–immunity cycle. Also, epigenetic pathways can impact both tumor cells as well as immune cells resulting in a negative impact on the anti-tumor immune response. For instance, DNA methylation-associated mutagenesis is the single most important source of genetic alterations, leading to neoantigen formation in most cancers including glioma (283, 284).

Several therapies aimed at targeting epigenetic pathways are being examined for their anti-glioma abilities. Several of these therapies target proteins that mediate histone modifications. Examples of these therapies include EZH2 inhibitors, DNA methyltransferase (DNMT) inhibitors, histone deacetylase (HDAC) inhibitors, mutant IDH inhibitors, and BET inhibitors (285). The enhancer of zeste homolog 1/2 (EZH1/2) is the main subunit of PRC2 responsible for the trimethylation of Histone H3 lysine 27 (H3K27me3), which controls stem cell and oncogenic gene expression programs (282). The H3K27 mutation has been shown to inhibit polycomb repressor complex 2 (PRC2) activity which leads to hypomethylation of H3K27 and expression of potential oncogenes (282). EZH2 overexpression is associated with poor GBM prognosis, and reducing levels of EZH2 expression in vivo resulted in a reduced tumor progression, which suggests the efficacy of EZH2 inhibitors as anti-glioma therapies (286, 287). EZH2 inhibitor, Tazemetostat, alone, and in conjunction with other therapies, is currently in clinical trials for treating pediatric glioma with EZH2, SMARCB1, or SMARCA4 mutations (ClinicalTrials.gov IDs NCT03213665, NCT03155620). These mutations affect gene expression via the regulation of chromatin remodeling.

DNA methylation is the most commonly studied epigenetic modification in cancer (285), and methylation signatures are included in glioma classification (288). Gliomas harboring mutant IDH1 display high levels of DNA hypermethylation in CpG rich domains, which are associated with increased tumor progression and altered gene expression (289, 290). Inhibitors of mutated IDH1/2 enzymes entered clinical trials and represent a novel drug class for targeted therapy of gliomas. These include AG-881, AG-120, and AG-221, all of which are being tested in preclinical and clinical settings. Preliminary results from Phase I clinical trials with IDH inhibitors demonstrated an objective response rate ranging from 31% to 40% with durable responses (>1 year) (291). To date, AG-120 showed the most clinically promising results as an orally administered, reversible, and highly selective small-molecule inhibitor of mutant IDH1/R132H cancers (292, 293).

Another group of drugs targeting the glioma methylation status are DNMT inhibitors, which are now being studied as potential anti-mIDH1 glioma therapies. DNMTs promote cancer generation by causing hypermethylation of tumor suppressor gene enhancers and promoter regions (18, 285). Early studies have shown anti-glioma efficacy of DNMT inhibitors in *vivo* and *in vitro* (294, 295). Despite the preclinical successes, a representative DNMT inhibitor, 5-aza-2′-deoxycytidine, has been shown to have minimal efficacy in early clinical trials (290). Recent studies showed a strong connection between epigenetics and cytokine production in tumor cells. One example is DNMT inhibition which can trick cancer cells into behaving as virus-infected cells, leading to activation of the interferon pathway (296, 297). In glioma, IL-6 promotes hypermethylation of the Sp1-binding site in the *miR142-3p* gene promoter, preventing binding of Sp1 and inhibiting miR-142-3p expression (298). These changes were shown to enhance the effectiveness of immune checkpoint inhibitors (296, 297). Moreover, multiple studies have shown that the PD-L1 level can be regulated by epigenetic mechanisms. For example, in IDH1 mutated glioma, we have shown that methylation in PD-L1 promoter negatively correlates with PD-L1 expression and prognosis (24).

Histone acetylation plays a role in gene expression. Whilst acetylation is generally associated with elevated transcription, deacetylated histones are generally associated with repressed genes (299). HDAC enzymes are differentially expressed in glioma and have been shown to play a role in glioma progression (18, 299). Several pre-clinical studies have shown an effective response for HDAC inhibitors via multiple mechanisms, including induction of tumor cell death, as well as increase radio-sensitivity, differentiation, and/or cell cycle arrest (300–302). Due to the promising results obtained from these studies, both Vorinostat and valproic acid, are currently being tested in clinical trials on gliomas as monotherapies and combinational therapies (18, 303–305). So far, results showed that HDAC inhibitor monotherapies are not sufficient as anti-glioma therapies, but they show promise in increasing the anti-glioma effects in combinational therapies (306).

Bromodomain and extra-terminal domain (BET) proteins are epigenetic chromatin readers that bind to acetyl marks of lysine residues to regulate gene expression (282, 285, 307). BET proteins were found to be associated with high expression of oncogenes (285, 307). BET inhibitors have been identified as possible therapies for GBM patients, as they have been found to inhibit GBM cell proliferation both *in vivo* and *in vitro* by hindering cell cycle progression and reducing oncogene expression (307, 308). Despite the promising preclinical findings, there are no clinical trials on BET inhibitors as a treatment for glioma patients.

Multiple therapeutics targeting epigenetic pathways (epidrugs) have been approved for cancer treatment which can affect the immune response. These were approved to treat hematopoietic malignancies such as T-cell lymphoma, multiple myeloma, and myelodysplastic syndromes (309, 310). Even though there is no clinical application of epidrugs targeting glioma, azemetostat, a KMT6A (EZH2) inhibitor, was approved for the treatment of epithelioid sarcoma, making it the first approved histone ‘writer’ inhibitor and the first epidrug to treat solid tumors (311). This demonstrates promising avenues of epidrugs to target solid tumors that have pronounced epigenetic dysregulation including glioma, which could in turn, enhance the immune response against these tumors.

### Inhibition of the Oncometabolite (R)-2-HG to Enhance Anti-Glioma Immunity

Mutation in the metabolic enzyme isocitrate dehydrogenase 1 (mIDH1) at active site residue R132H occur in ~20-25% of infiltrative gliomas (40, 312, 313). The mutation leads to gain-of-function catalytic activity that converts α-ketoglutarate (αKG) to the onco-metabolite (R)-2-hydroxyglutarate ((R)-2-HG) (40, 314, 315). (R)-2-HG competitively inhibits histone demethylating enzymes ten-eleven translocation methylcytosine dioxygenases (TETs) and lysine-specific demethylases (KDMs) (289, 316). Inhibiting demethylation increases DNA and histone methylation, altering the epigenome, resulting in changes in the tumor transcriptome (289, 316). Although studies have shown that small molecule inhibitors targeting IDH1-R132H have been effective in impairing tumor progression as monotherapy in pre-clinical models (317), in phase I clinical trials they have not been effective as monotherapies (NCT02381886). We have previously shown that mIDH-R132H, in the context of *ATRX* and *TP53* inactivation, epigenetically reprograms gene regions corresponding to DNA repair proteins in human and murine glioma cell cultures (44). Treatment with AGI-5918, a small molecule inhibitor prior to radiotherapy, downregulated DNA repair gene expression, thus making the tumor cells radiosensitive. These results highlight the need for a combinatorial treatment strategy to effectively impede mIDH1 progression.

The onco-metabolite (R)-2-HG has been shown to repress expression of key immune regulatory genes, such as CCL2, CXCL-2 and C5-a, which are primarily involved in mediating lymphocytes’ trafficking to the mIDH1 glioma TME (94). Recently, one study demonstrated that combination of PD-1 inhibition and the mIDH1 inhibitor BAY1436032 extended the survival of mice implanted with GL261-IDH1R132H glioma cells by overriding the immune suppressive environment mediated by (R)-2-HG (318).

We recently demonstrated that in genetically engineered mIDH1 mouse gliomas, resembling human mutant IDH1 astrocytoma, (R)-2-HG inhibition in combination with SOC increased the infiltration of DCs and anti-tumor specific T cells in the TME, while decreasing the infiltration of immunosuppressive MDSCs, Tregs, and M2 macrophages compared to saline treated mice (24). We also observed that mIDH1 glioma cells exhibit lower levels of PD-L1 expression (24). In response to (R)-2-HG inhibition, PD-L1 expression levels on mIDH1 glioma cells significantly increased to those observed in wild type IDH gliomas (24). Numerous preclinical solid tumor models have demonstrated that the immune checkpoint blockade of PD-1/PD-L1 interaction prevents T cell exhaustion, resulting in enhanced anti-tumor immune activity and improved MS (9, 319, 320). We previously demonstrated that PD-L1 checkpoint blockade as monotherapy elicited a small increase in MS in mice bearing syngeneic glioma, with only a few long-term survivors (9). However, immune-checkpoint blockade used as monotherapy has failed in Phase III clinical trials to improve OS of patients with glioma (12). We observed that IDH1-R132H inhibition used in combination with SOC and anti-PD-L1 immune checkpoint blockade increased the frequency of tumor-specific cytotoxic CD8+ T cells and IFN-γ release within the TME (24). Strikingly, long-term survivors from IDH1-R132H inhibition in combination with SOC and anti-PD-L1 immune checkpoint treatment group remained tumor-free post mIDH1 glioma rechallenging in the contralateral hemisphere, indicating the development of anti-mIDH1 glioma immunological memory (24). This is a critical factor in determining the success of immune-therapeutic approaches in gliomas. A robust anti-tumor T cell response and the presence of anti-glioma immunological memory are required to eradicate any remnant tumor cells post-surgery and prevent recurrence.

Collectively, upon metabolic reprogramming it is possible to achieve anti-mIDH1 glioma immunity. The precise elucidation of the immune pathways affected by (R)-2-HG will lead to an understanding of the underlying biological processes and will provide better therapeutic approaches for mIDH1 glioma patients.

## Future Prospects and Conclusions

The path for immunotherapies against glioma has started more than a decade ago and the lack of sustained clinical beneficial outcomes demonstrates the challenge that this tumor represents (12, 17, 79, 84, 249). Malignant gliomas are tumors intrinsically difficult to target by immunotherapies due to their heterogeneity, their immunosuppressive TME and the particular cross-talk of the CNS and the immune system (6, 11). However, as tumor recurrence occurs almost always in glioma patients, since these tumors are virtually impossible to completely resect due to their infiltrative nature, anti-glioma immunological memory would be highly beneficial desirable for these patients. Also, because the immunosuppressive TME is related to glioma aggressiveness, trying to counteract this milieu represents an appealing idea for oncologists and researchers.

**Figure 2:**
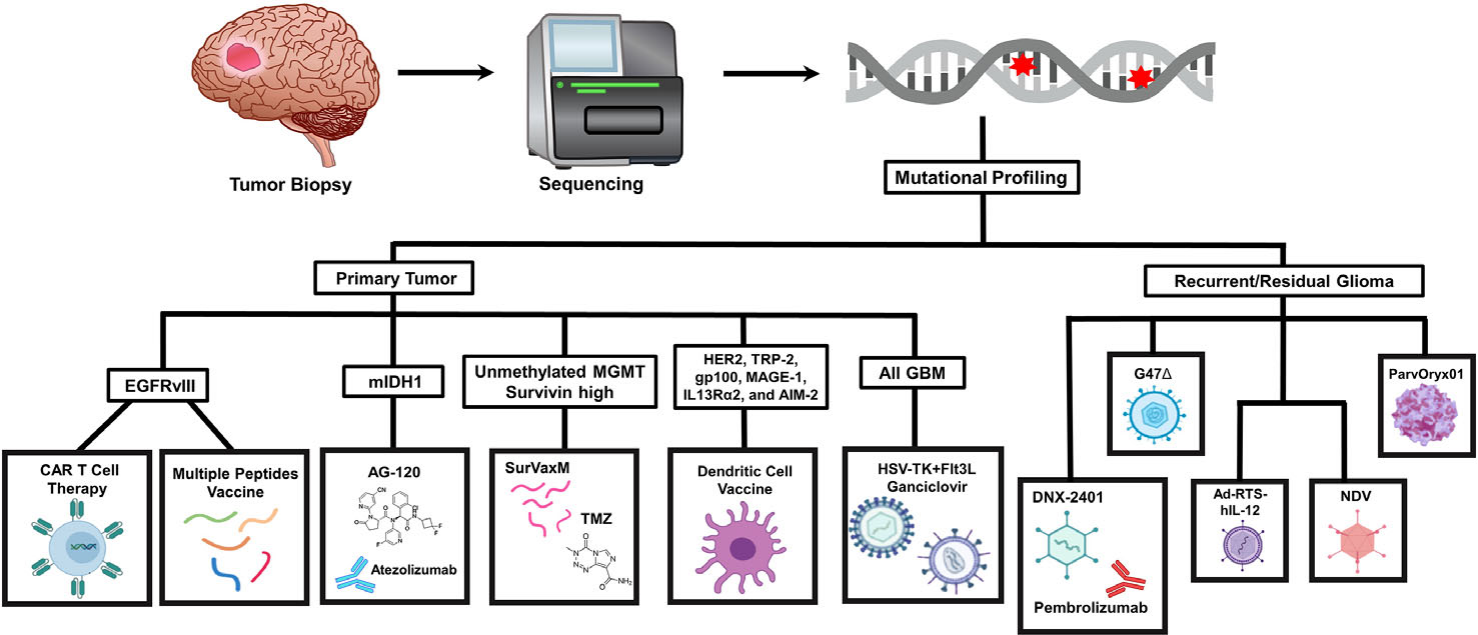
Genomic sequencing of glioma patient tumor biopsies could guide the immunotherapy strategies selected based on the mutational profile and clinical status. Tumor biopsies obtained by surgical resection undergo high-throughput genomic sequencing to identify mutations present within the cancer cells. The genetic lesions detected could be used as a decision factor to select which immunotherapy to choose. Patients with primary tumors that express specific tumor antigens can undergo a variety of immune-based treatments that address these driver mutations directly and could be combined with chemotherapeutic agents and other approaches, such as immune checkpoint inhibitors. Patients that present with recurrent or residual glioma could also be treated with gene therapy or virotherapy-mediated approaches that directly target the glioma cells to trigger immune-stimulatory mechanisms.

Research in immunotherapies against glioma developed in parallel with the broadening of our understanding regarding the molecular landscape associated to different types of glioma. These studies demonstrated that entities before defined under the same WHO group, were not as homogeneous as they were thought to be (38, 40, 321). The genetic and epigenetic data gathered so far enabled the classification of gliomas in terms of their intrinsic characteristics, in combination with the histological features, and unveiled how complex these tumors are. Also, with the advent of state-of-the-art technologies, such as scRNA-Seq, it was realized not only that gliomas have intra-tumor heterogeneity, but also, that this state could fluctuate, for example, depending on the stage of the treatment at which the biopsy is taken. Ideally, in the future, the advancements in both, sequencing methodologies and immunotherapeutic strategies, will be combined to design and apply more targeted therapies for glioma patients (**Figure 2**).

This overwhelming amount of information allowed the development of more sophisticated murine models for the pre-clinical testing of immune-mediated therapies for glioma. Mouse models that recapitulate the human disease, with animals harboring brain tumors encoding for the specific genetic and epigenetic alterations described, have been generated with techniques, such as the Sleeping Beauty method, that enable the concomitant study of the surrounding immune response.

Even though these advancements represented a milestone in the exploration of immunotherapies against glioma, the translation of pre-clinical findings to the clinical setting has not yielded consistent or sustained beneficial outcomes for patients. This drawback reveals that animal models need to be further adjusted to the genetics/biology of these tumors and that we should be cautious about generalizing the potential clinical response for a particular immune-mediated therapy. So far, the data gathered from clinical trials and the information obtained in pre-clinical models have been useful in demonstrating that the molecular features of glioma influence the anti-tumor immune response and the clinical consequences of the administration of an immunotherapy.

Despite the lack of a substantial benefit for glioma patients treated with immunotherapies, the medical-research community has learnt important lessons from these pitfalls. Currently, we know that the immune-mediated approach to treat glioma should be combinational, not only by considering more than one TAA or TSA to target, but also by integrating different immunotherapeutic strategies. For example, DC vaccines could be combined with ICI and OV therapy, to enhance the development of adaptive anti-tumor immunity. The possibility of combinational therapies is encouraging, as we have been capable of developing different immune-mediated strategies against this tumor. However, it rises the complexity of tumor treatment, as the number of combinations for drug doses, times and routes for drug administration increases exponentially.

The great advancements in the immune-mediated approaches for glioma therapy and the development of BBB penetrating and tumor-targeted ways of drug administration in the pre-clinical setting has demonstrated that the research community is capable of designing new alternatives to overcome the challenges that this type of tumor presents. We believe that the key for the success of immunotherapies against glioma resides in the deep understanding of the biology of this tumor and in the precise combination of diverse therapeutic approaches. It is important to carefully revise the clinical trial results and to compare them with the pre-clinical data, in order to learn from the failures and generate better animal models.

We hope that the data discussed in this review highlight the importance of taking into account the molecular features of gliomas when considering immunotherapies and that it will shed light on the aspects that we still need to tackle to successfully harness the immune system against these tumors.

## Author Contributions

All the authors contributed to the writing of the manuscript. MC, AS, and NG prepared **Figure 1** and tables. SC prepared **Figure 2**. All authors contributed to the article and approved the submitted version.

## Funding

This work was supported by NIH/NINDS Grants, R37-NS094804, R01-NS105556 and 1R21NS107894 to MGC; NIH/NINDS Grants R01-NS076991, and R01-NS096756 to P.R.L.; NIH/NIBIB: R01-EB022563 grant to MGC, PL and JJM; the Department of Neurosurgery, the Rogel Cancer Center, Program in Cancer Hematopoiesis and Immunology (CHI), the ChadTough Foundation, Pediatric Brain Tumor Foundation, and Leah’s Happy Hearts to MGC. and PL; RNA Biomedicine Grant F046166, Forbes Foundation Grant, University of Michigan Medical School, Rogel Cancer Center Scholar, University of Michigan Medical School to M.G.C.; T32 CA009676-26 Cancer Biology Training Grant to MAl; UL1 TR002240 for the Michigan Institute for Clinical and Health Research (MICHR), Postdoctoral Translational Scholars Program (PTSP), Project F049768 to AC and American Brain Tumor Association Basic Research Fellowship “in Memory of Bruce and Brian Jackson” to MG-F.

## Conflict of Interest

The authors declare that the research was conducted in the absence of any commercial or financial relationships that could be construed as a potential conflict of interest.

## Abbreviations

CNS, Central nervous system; GBM, Glioblastoma; HGG, High grade glioma; ICI, Immune checkpoint inhibitors; LGG, Low grade glioma; MDSC Myeloid-derived suppre,ssor cells; MS, Median survival; OS, Overall Survival; OVs. Oncolytic Viruses; PFS, Progression Free Survival; PBMCs, peripheral blood mononuclear cells; SOC, Standard of care; TAA, Tumor-associated antigen; TILs, Tumor-infiltrating lymphocytes; TME, Tumor microenvironment; TMZ, Temozolomide; Tregs, Regulatory T cells; TSA, Tumor-specific antigen.
